# The Effect of Depression and Hopelessness on Suicidal Risk in Young People: The Mediating Role of Impulsivity

**DOI:** 10.32872/cpe.11331

**Published:** 2025-05-28

**Authors:** Anyerson Stiths Gómez-Tabares, Olber Eduardo Arango-Tobón, César Núñez, Gastón Adolfo Zapata Lesmes

**Affiliations:** 1 Faculty of Social Sciences, Health and Wellness, Universidad Católica Luis Amigó, Medellín, Colombia; 2 Psychology Program, Faculty of Social and Human Sciences, Universidad de Medellín, Medellín, Colombia; 3 Corporación APICSA, Medellín, Colombia; 4 Faculty of Social Sciences, Health and Wellness, Universidad Católica Luis Amigó, Manizales, Colombia; Philipps-University of Marburg, Marburg, Germany

**Keywords:** suicide, impulsivity, depression, emotions, risk

## Abstract

**Background:**

Previous studies have documented that depression and hopelessness predict higher suicide risk in young people. However, the psychological mechanisms that may mediate these associations are unknown. The aim of this study was to analyze the effects of depression, hopelessness, and impulsivity on suicidal attempts and risk, and to explore the mediating role of impulsivity in these associations.

**Method:**

A total of 1,645 young people participated with a mean age of 21.604 years (*SD* = 3.22) (68.8% female and 31.2% male). A sociodemographic form was applied to explore suicide attempts in the last year (SA), the Plutchik Suicide Risk (SR), Beck Hopelessness (BHS), Barratt Impulsivity (BIS), and Beck Depression Inventory (BDI) scales. Direct correlations were found among BHS, BDI, BIS, SA, and SR.

**Results:**

The binary regression model showed that the variables BHS, BDI, and BIS explained between 33% and 49% of the variance of suicidal risk and 16% of the variance of suicide attempts. Structural equation analysis showed that impulsivity mediated the associations between depression, hopelessness, and suicidal risk, on the one hand, and mediated the associations between depression and suicide attempt, on the other hand, whose total direct and indirect effects were statistically significant.

**Conclusion:**

The findings emphasize the importance of impulsivity as the mechanism influencing interactions between mood indicators and suicidal behavior in young populations.

## Highlights

- Depression and hopelessness have been identified as significant predictors of suicidal behavior in young people.
- The effect of depression and hopelessness on suicidal behavior is mediated by impulsivity.
- The effect of depression, hopelessness, and impulsivity on suicidal behavior showed invariance by sex (male/female).

According to the Global Health Estimates Report (WHO, 2021), suicide is the fourth leading cause of death worldwide in young people aged 15-19 years. Previous attempts have been reported to be the most important risk factor for suicide in the general population. According to the Institute of Legal and Forensic Medicine in Colombia 2,952 suicides were registered, and the annual rate corresponds to 6,16 cases per 100,000 inhabitants in 2022 (National Institute of Legal Medicine and Forensic Sciences, 2023).

Several current systematic reviews have identified a broad set of risk factors associated with suicide, including social isolation, mental disorders, alcohol abuse, family-related events, information processing styles, and deficits in neuropsychological functions (Ati et al., 2021; González Sancho & Picado Cortés, 2020; Hernández-Bello et al., 2020). Nevertheless, the role of personality factors, such as impulsivity, remains under investigation. The current evidence is inconsistent regarding the mediating role of impulsivity among depression, hopelessness, suicide attempts, and suicidal behavior.

Research on cognitive processing has found that hopelessness plays an important role in suicidal risk and behavior and has a mediating role between depressive symptoms and suicide attempts in adults and youth (Choi & Shin, 2023; Dat et al., 2021).

Horwitz et al. (2017) found that hopelessness is a longitudinal predictor of depression in youth at high suicidal risk. Their study suggests that lack of positive expectations is the most significant predictor of depression and future suicidal behavior, as hopelessness magnifies depressive symptoms, which in turn increases the likelihood of suicide, especially when combined with loss of social connectedness and loneliness.

Indeed, negative circumstances related to stressors in various roles, such as family conflicts, lack of support networks, or social pressure, can influence the development of suicidal ideation (Dutton et al., 2013; Puzia et al., 2014). These factors align with Joiner's (2005) theory, which posits that the perception of being a burden to others (e.g., family, friends, or society) is associated with both an increased risk of suicide and the acquired capability for suicide.

In this context, path analyses proposed by Dat et al. (2021) in a sample of 322 young college students demonstrated that hopelessness functions as a mediator of the effect of self-esteem and social anxiety, intensifying depressive symptoms and suicidal ideation. Additional studies have indicated that hopelessness is a predictor of depression (Choi & Shin, 2023), and both psychological factors would strongly predict suicidal risk in young populations (Gómez-Tabares et al., 2024; Núñez et al., 2023).

Some studies have approached risk factors from a direct effect and mediation approach to suicidal behavior, noting the importance of recognizing the interaction of these elements in the mechanism that predicts suicide. Similarly, Wang et al. (2015) found that hopelessness, depression, and impulsivity contribute significantly to the development of suicidal risk and behavior, but such contribution depends on how these factors interact with each other. In their study, they concluded that hopelessness mediated by the severity of depression has an indirect effect on suicidal ideation because hopelessness is not a direct cause of suicidal behavior and only gains strength when mediated by depressive symptomatology. This mechanism of direct and indirect effects is dependent on high or moderate levels of impulsivity in patients with depression since these patients with greater impulsivity are more likely to increase suicide attempts and display suicidal behavior in general.

Therefore, an important construct in the interaction of suicide risk factors is impulsivity. Current evidence suggests that impulsivity is a significant component of suicidal behavior and plays an important role in the transition from suicidal ideation to suicide attempt (Beach et al., 2022; Cole et al., 2019). Zhang et al. (2022), in a community sample of 480 college students, examined the effect of impulsivity on suicidal ideation by depression. The results indicated that impulsivity had an indirect but mediating effect on depression and concluded that the higher the students' impulsivity, the stronger the predictive effect of depression on suicidal ideation.

Other studies indicate that people with higher impulsivity scores report significantly more suicide attempts than patients with lower impulsivity scores (Mann et al., 1999). However, in a meta-analysis conducted by Anestis et al. (2014), the central role of impulsivity in suicide attempts and risk is questioned, noting that impulsivity is a distal risk factor that modulates the effect of more direct factors such as precipitating experiences that increase suicide in individuals.

Literature has indicated that depression and hopelessness may interact with impulsivity to generate an increased risk for suicidal attempts and behaviors (Arango-Tobón et al., 2021; Dumais et al., 2005; Swann et al., 2008). Arango-Tobón et al. (2021) points out that impulsivity is a mediator between depression and suicidal behavior and acts as a trigger and determinant of suicide in young people.

Given the above, further evidence on the mediating role of impulsivity between depression and hopelessness may generate better assessment processes for the identification of suicidal behaviors and better ways to intervene early in short-term suicide risk indicators during youth. The present study aimed to explore whether impulsivity plays a mediating role between depression and hopelessness concerning suicide attempts and risk. We hypothesized that 1) Both depression and hopelessness have significant direct effects on suicide attempts and risk, but 2) Impulsivity as a mediator between depression and hopelessness amplifies their direct or indirect effects on suicide attempts and risk in young people.

## Method

### Participants

A stratified random probability sampling by academic semesters from three private universities in Manizales and Medellín (Colombia) was used. A total of 2,580 students were invited to participate voluntarily in this study. A total 935 people did not participate in the study for the following reasons: minors (<18 years of age) who did not provide informed parental consent (*n* = 92), were absent on the day of data collection, or did not want to participate in the study (*n* = 175), did not complete all the questionnaires administered or left more than five items in a row unanswered in one or more instruments (*n* = 668).

The main inclusion criterion is that they were young people between the ages of 18 and 30 since the highest suicide rates in Colombia occur among young people aged 18 and 19 (10.43 per 100,000 inhabitants), 20 to 24 (9.98 per 100,000 inhabitants) and 25 to 29 (8.16 per 100,000 inhabitants) (Instituto Nacional de Salud, 2023). In addition, the highest incidence of suicide attempts also occurs in the young population and has increased systematically in the last five years in the cities of Manizales and Medellin, Colombia. Furthermore, the highest incidence of suicide attempts is observed in the young population, with a notable increase over the past five years in the cities of Manizales and Medellín, Colombia.

The final sample consisted of 1,645 young people attending three private universities in two Colombian cities, Manizales (*n* = 992, 60.3%), and Medellín (*n* = 653, 39.7%). In terms of sex, 1,131 were women (68.8%) and 514 were men (31.2%). The mean age was 21.604 years (*SD* = 3.22). Regarding the socioeconomic level, 24.4% belonged to the lower level, 65.5% to the middle level, and 10.2% to the upper level. 62.2% reported not having a partner, 30.9% reported having a partner relationship, 3.5% lived with their partner, 2.1% were married and 0.9% reported no information. Participants from both cities were from urban areas and did not differ in terms of socioeconomic status (*z* = -0.894, *p* = .371). There were no indigenous or rural populations. The results section describes the mental health indicators of the youth reported from the instruments.

### Instruments

#### Ad Hoc Sociodemographic Form

This self-administered form collected data on participants' age, sex, place of residence, socioeconomic status, history of suicide attempts, and number of such attempts. The variable corresponding to suicide attempts was obtained from self-reports of at least one attempt in the past year, assessed using a dichotomous response format (yes/no). Responses were coded numerically, assigning a value of 0 to a no response and 1 to a yes response.

#### Plutchik Suicide Risk Scale (SR)

It is a Likert-type instrument designed to assess the risk of suicide attempts (Plutchik & Van Praag, 1989). It includes 15 items, each with dichotomous response options (Yes/No). Affirmative responses are scored with one point, resulting in a maximum score of 15. A score above 6 suggests the presence of suicidal risk (Rubio et al., 1998). The scale is frequently used in research with young population in Colombia (Gómez-Tabares, 2020; Gómez-Tabares et al., 2024; Núñez et al., 2023; Suárez-Colorado et al., 2019), demonstrating internal consistency with Cronbach's alpha values above 0.75. For this study, internal consistency between 0.77 (Cronbach's alpha) and 0.82 (McDonald's Omega) was evidenced. Confirmatory factor analysis (CFA) showed that the scale was a good fit for a unidimensional model of suicide risk (GFI = 0.972, AGFI = 0.960, RMSEA = 0.043).

#### Beck Hopelessness Scale (BHS)

It is a screening instrument to detect feelings of hopelessness associated with depression and suicide risk. It was developed by Beck et al. (1974) and consists of 20 dichotomous (true/false) items reflecting cognitive and emotional components of hopelessness. Scores range from 0 to 20, with higher scores indicating greater severity of hopelessness. The scale allows the severity of hopelessness to be classified as minimal (0 to 3), mild (4 to 8), moderate (9 to 14), and severe (15 to 20). Validation studies conducted with the Colombian population report a Cronbach's alpha between 0.82 and 0.93, test-retest reliability coefficients between 0.60 and 0.69 (Rueda-Jaimes et al., 2018), and optimal fit for a unidimensional structure (CFI = 0.99, RMSEA = 0.03) (Pineda-Roa et al., 2024). The internal consistency for this study was .84 (Cronbach's alpha) and .88 (McDonald's Omega). Confirmatory factor analysis was also conducted and indicated that the data were consistent with a unidimensional model (GFI = 0.973, AGFI = 0.964, RMSEA = 0.033).

#### Beck Depression Inventory (BDI)

It is a 21-item self-report measure designed to assess the severity of depressive symptoms (Beck et al., 1979). It assesses affective, cognitive, physiological, and behavioral aspects of depression. Each item is scored on a scale of 0 to 3, with a total score ranging from 0 to 63. Higher scores indicate greater severity. The inventory allows the classification of depressive symptoms into minimal (0 to 9), mild (10 to 16), moderate (17 to 29), and severe (30 to 63). Studies conducted with university students in Colombia have shown internal consistency with Cronbach's alpha values between 0.88 and 0.92 (Arango-Tobón et al., 2021; Núñez et al., 2023). In this study, internal consistency was 0.89 (Cronbach's alpha) and 0.91 (McDonald's Omega). Confirmatory factor analysis supported a unidimensional structure with good fit indices (GFI = 0.967, AGFI = 0.959, RMSEA = 0.035).

#### Barratt Impulsivity Scale, v. 11 (BIS-11)

This is a self-report Likert-type scale designed to assess impulsivity as a behavioral trait (Patton et al., 1995). The linguistic equivalence of the BIS-11 has been demonstrated for use in Spanish-speaking population (Oquendo et al., 2001). The scale consists of 30 items on a 4-point scale (rarely or never, occasionally, often, and always or almost always). Studies conducted with Colombian adolescent and adult populations have demonstrated acceptable internal consistency for the total scale score, reporting Cronbach's alpha values between 0.75 (Urrego Barbosa et al., 2017) and 0.795 (Chachín Pinzón et al., 2019). Additionally, Stanford et al. (2009) highlighted the use of a score of 74 in psychological studies to identify impulsivity. The internal consistency for this study was 0.75 (Cronbach's alpha) and 0.79 (McDonald's omega). Confirmatory factor analysis supported a unidimensional structure with acceptable goodness-of-fit indices (GFI = 0.943, AGFI = 0.931, RMSEA = 0.040).

### Procedure and Ethical Aspects

The recruitment of participants did not involve any specific clinical criterion and was carried out through the modality of subjects available in the classrooms, according to the stratification by academic semester of three university institutions in the cities of Manizales and Medellin, Colombia. After the application of the informed consent form and on a voluntary basis, the young people filled out the instruments manually with pencil and paper.

The study was approved by the Ethics Committee of the Universidad Católica Luis Amigó and the Corporación Coetika, Manizales, Colombia. It was research without risk for the participants (Resolution 8430 of 1993) and was ethically oriented in Law 1090 of the Colombian College of Psychologists regarding the exercise of research. Law 1266 of 2008; Law 1581 of 2012 and Decree 1377 of 2013 regarding the handling of personal data were taken into account when considering the criteria for the collection, handling, and special protection of personal data and the use of information for academic research purposes. Although the sampling did not include specific clinical criteria, young people who showed an indicator of suicidal risk according to the Plutchik scale were referred to the University Welfare Service for counseling and psychological support.

### Data Analysis

Data analysis was performed using SPSS version 25.0. First, a descriptive analysis of the socio-demographic characteristics of the sample was carried out. The internal consistency of the instruments was confirmed using Cronbach's alpha and McDonald's omega coefficients. The instruments were also tested for fit to a unidimensional structure from confirmatory factor analysis (CFA) (Byrne, 2016). Descriptive and frequency statistics were calculated for the suicide risk, suicide attempt, depression, hopelessness and impulsivity variables. Kolmogorov-Smirnov and chi-squared (χ^2^) tests were used to assess the distribution of the data, which showed that they were not normally distributed.

The Mann-Whitney *U* test was used to compare scores for suicide risk and attempt, depression, hopelessness and impulsivity by sex on the one hand, and suicide risk factor on the other. The *p*-value was reported and effect sizes were calculated for these comparisons. The eta-squared (η^2^) was used as a measure of effect size (small (0.01), medium (0.06) and large (0.14) (Fritz et al., 2012). Spearman's rho was used to assess correlations between the variables of depression, hopelessness and impulsivity and suicide risk and attempt.

Two path analysis models were then developed to estimate the standardized direct and indirect effects between the variables. These models were analyzed using generalized least squares. Bootstrapping with a 95% confidence interval was used to estimate total, direct, and standardized indirect effects (Byrne, 2016).

Model fit was assessed by probability χ^2^ (*p ≥* .05), χ^2^*/df* (values < 3), and additional fit indices including comparative fit index (CFI ≥ 0.90), incremental fit index (IFI ≥ 0.90), Tucker-Lewis index (TLI ≥ 0. 90), goodness of fit index (GFI ≥ 0.90), adjusted goodness of fit index (AGFI ≥ 0.90), normed fit index (NFI ≥ 0.90), and root mean square error of approximation (RMSEA ≤ 0.08) (Byrne, 2016; Schermelleh-Engel et al., 2003). Finally, a multigroup analysis was performed to test the invariance of the structural model between sexes (male/female). The ΔCFI criterion was used to assess model equivalence, with a change equal to or less than 0.01 (ΔCFI ≤ 0.01) supporting invariance (Cheung & Rensvold, 2002). Path and multigroup analyses were performed in Amos v. 24.0 software.

## Results

Regarding the most relevant mental health indicators reported, a suicide risk factor was found in 26.7% of the young people and a history of a previous suicide attempt in 13.2%. It was also found that 36% of the young people reported some symptoms of depression and 27.2% reported indicators of hopelessness (Supplementary Materials, Table 1). We found that women had higher scores than men in suicidal risk and depression. When assessing the effect size of significant sex differences, a small effect size was identified. No differences were found between men and women in suicide attempts, hopelessness, and impulsivity (Supplementary Materials, Table 2).

A comparative analysis of psychological variables as a function of suicide risk and attempt was performed (see Table 1). The young people who presented a suicide risk factor showed higher scores in depression, hopelessness, and impulsivity compared to the group without risk. Additionally, youth who reported any suicide attempt in the last year presented higher scores in suicidal risk, depression, hopelessness, and impulsivity compared to the group with no history of attempts. All differences were statistically significant (*p* < .001).

**Table 1:** Differences According to Suicide Risk and Attempt and the Variables of Depression, Hopelessness, and Impulsivity

Variable	*M*	*SD*	*AR*	*Mdn*	*M*	*SD*	*AR*	*Mdn*	*z*	*p*	η^2^
	Without risk				With risk				Test statistic		
Suicide attempt	1.02	0.15	734.29	1.00	1.43	0.50	1065.93	1.00	-21.385	< .001	0.278
Depression	6.11	5.42	666.59	5.00	17.38	9.59	1251.35	17.00	-22.136	< .001	0.298
Hopelessness	2.03	2.11	705.20	2.00	5.49	4.48	1145.60	4.00	-16.872	< .001	0.173
Impulsivity	47.48	12.37	715.19	46.00	59.41	13.55	1118.25	60.00	-15.237	< .001	0.141
	Without suicide attempt				With suicide attempt				Test statistic		
Suicide risk	3.08	2.46	733.72	3.0	7.76	2.23	1410.53	8.0	-19.683	< .001	0.236
Depression	8.12	7.64	772.63	6.0	15.70	10.24	1154.44	15.0	-11.050	< .001	0.074
Hopelessness	2.68	2.99	789.14	2.0	4.80	4.48	1045.85	3.0	-7.519	< .001	0.034
Impulsivity	49.52	13.23	785.47	48.0	58.29	14.65	1069.99	58.0	-8.223	< .001	0.041

*Note. M* = Mean; *SD* = Standard Deviation; *Mdn* = Median; *AR* = Average Range; η^2^ = Eta Square.

Table 2 shows the results of the correlational analysis of the various study variables using Spearman's *Rho* coefficient. Suicide risk and attempt correlated directly and significantly (*p* < .001) with depression, hopelessness, and impulsivity.

**Table 2:** Spearman Correlation Coefficient (Rho) Between Suicide Risk and Attempt and the Variables of Depression, Hopelessness, and Impulsivity

Variable	1	2	3	4	5
1. Suicide risk	–	0.485**	0.679**	0.461**	0.474**
2. Suicide attempt		–	0.273**	0.185**	0.203**
3. Depression			–	0.516**	0.432**
4. Hopelessness				–	0.362**
5. Impulsivity					–

**p < .001.

Table 3 presents two binary logistic regression models using the input method, in order to identify the role of depression, hopelessness, and impulsivity variables on the variance of suicide risk and attempt. The first model used the suicide risk factor and the second the history of a suicide attempt as a dependent variable. Depression, hopelessness, and impulsivity were the independent variables. Both the first model, as assessed by the Hosmer-Lemeshow test (χ^2^ = 7.830, *df* = 8, *p* = .750), and the second model (χ^2^ = 12.744, *df* = 8, *p* = .521), presented good indicators of goodness of fit.

**Table 3:** Binary Logistic Regression Analysis: Suicide Risk Factor and Suicide Attempt as Dependent Variables

Variable	β	*SE*	χ^2^ Wald	*df*	*p*	*OR*	95% CI for *OR*	
							*LL*	*UL*
Model 1. Suicide risk factor as a dependent variable								
Depression	0.153	0.012	169.694	1	< .001	1.166	1.139	1.193
Hopelessness	0.130	0.027	22.395	1	< .001	1.138	1.079	1.201
Impulsivity	0.036	0.006	36.967	1	< .001	1.036	1.025	1.048
Model 2. Suicide attempt history as a dependent variable								
Depression	0.069	0.011	41.677	1	< .001	1.072	1.050	1.095
Hopelessness	0.006	0.025	0.065	1	0.799	1.007	0.958	1.058
Impulsivity	0.025	0.006	16.511	1	< .001	1.025	1.013	1.038

The first model showed that the independent variables explained between 33% (*R^2^* Cox and Snell = 0.334) and 49% (*R*^2^ Nagelkerke = 0.486) of the suicide risk factor and the second model explained between 8% (*R*^2^ Cox and Snell = 0.084) and 16% (*R*^2^ Nagelkerke = 0.156) of the variation in a suicide attempt.

The odds ratios (*OR*) show how much the probability of belonging to the suicide risk and attempt categories increases according to the variance of the independent variables. In Model 1, depression, hopelessness, and impulsivity increased the suicide risk factor. For Model 2, depression and impulsivity contributed a significant effect that increased the probability of suicide attempts. Hopelessness did not contribute a significant effect on a suicide attempt.

To establish the total, direct and indirect standardized effects of the independent variables on suicide risk and attempt, two structural equation models were estimated using the generalized least squares method (Byrne, 2016). In the first model, the direct effect of the dependent variables depression, hopelessness, and impulsivity on suicide risk and suicide attempt was estimated, but it did not yield good goodness-of-fit indicators at the χ^2^ probability level and the RMSEA indicator, so the model was re-specified to improve goodness-of-fit indicators and establish patterns of association with mediating variables. In Model 2, impulsivity was used as a mediating variable between depression, hopelessness, suicidal risk, and suicide attempts. Additionally, suicide attempt was placed as a mediator between impulsivity and suicidal risk. This model obtained better goodness-of-fit indicators (see Table 4). Additionally, a sex invariance analysis was performed in order to corroborate whether Model 2 is equivalent between males and females (see Table 4).

**Table 4:** Goodness-of-Fit Statistics of Structural Models for Predicting Suicide Risk and Suicide Attempt

	χ^2^	*df*	χ^2^*/df*	IFI	CFI	NFI	TLI	GFI	AGFI	RMSEA
Model										
Model 1	15.618	2	7.809	0.984	0.984	0.982	0.921	0.996	0.971	0.064
Model 2	1.010*	1	1.010	1.000	1.000	0.999	1.000	0.999	0.996	0.002
Invariance by sex (male/female) of Model 2										
Without restrictions	1.020*	2	0.510	1.001	1.000	0.999	1.011	0.999	0.996	0.000
Structural weights	6.933*	10	0.693	1.004	1.000	0.992	1.007	0.998	0.995	0.000
Structural covariance	8.768*	13	0.674	1.005	1.000	0.990	1.008	0.998	0.995	0.000
Structural waste	10.070*	16	0.629	1.007	1.000	0.988	1.009	0.998	0.995	0.000

*p ≥ .05.

Table 4 shows the goodness-of-fit indices of Model 2, with different levels of restriction to assess sex invariance (male/female). When comparing the differences in CFI, values of ΔCFI *≤* 0.010 were observed, demonstrating the sex invariance of the proposed model.

**Figure 1:**
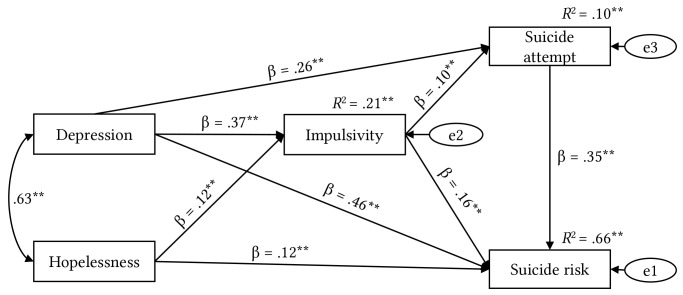
Structural Equation Modeling of Suicide Risk and Attempt and the Mediating Role of Impulsivity **p < .001.

Figure 1 shows that the variables: depression, hopelessness, impulsivity, and previous suicide attempt explained 66% (*R*^2^ = 0.655, *p* < .001) of the variation in suicidal risk. Depression and impulsivity variables contributed significant effects on suicide attempts (*R*^2^ = 0.101, *p* < .001), and depression and hopelessness variables explained 21% (*R*^2^ = 0.206, *p* < .001) of the variation in impulsivity. Likewise, it was found that impulsivity mediated the association between depression, hopelessness, and suicidal risk, on the one hand, and mediated the association between depression and suicide attempt, on the other hand, whose total direct and indirect effects were statistically significant (*p* < .001) (see Table 5).

**Table 5:** Standardized Total, Direct and Indirect Effects of Predictor Variables on Response Variables

Effect	Impulsivity (Mediator)			Suicide attempt (Mediator)			Suicide risk		
	Value β	CI 95%		Value β	CI 95%		Value β	CI 95%	
		*LL*	*UL*		*LL*	*UL*		*LL*	*UL*
Hopelessness									
Total	0.122**	0.066	0.179	0.012**	0.005	0.022	0.141**	0.100	0.181
Direct	0.122**	0.066	0.179	–	–	–	0.116**	0.077	0.156
Indirect	–	–	–	0.012**	0.005	0.022	0.024**	0.013	0.037
Depression									
Total	0.367**	0.311	0.420	0.297**	0.241	0.351	0.621**	0.582	0.659
Direct	0.367**	0.311	0.420	0.260**	0.199	0.320	0.458**	0.416	0.499
Indirect	–	–	–	0.037**	0.017	0.058	0.163**	0.138	0.189
Impulsivity									
Total	–	–	–	0.101**	0.046	0.154	0.199**	0.162	0.236
Direct	–	–	–	0.101**	0.046	0.154	0.165**	0.131	0.199
Indirect	–	–	–	–	–	–	0.035**	0.016	0.054
Suicide attempt									
Total	–	–	–	–	–	–	0.346**	0.311	0.380
Direct	–	–	–	–	–	–	0.346**	0.311	0.380
Indirect	–	–	–	–	–	–	–	–	–

**p < .001.

Table 5 shows the total, direct and indirect standardized effects of the independent, mediating and dependent variables. Depression contributed the largest total effect on impulsivity, suicide attempt, and suicidal risk. Likewise, impulsivity is a mediator between depression and hopelessness, whose indirect effects were significant (*p* < .001). Since all direct and indirect effects are statistically significant, it can be concluded that the mediation effect of the impulsivity and suicide attempt variables is partial.

## Discussion

The aim of the present study was to examine the role of impulsivity relative to depression and hopelessness in suicidal attempts and risk. We hypothesized that 1) depression, hopelessness, and impulsivity have direct effects on suicide risk and attempts, but the mechanism of influence among these predictors may not be the same as when analyzed together, and, therefore, 2) impulsivity may play a mediating role between depression and hopelessness and this role amplifies their direct or indirect effects on suicidal attempts and risk in youth.

According to the results of our study, it is clear that depression, hopelessness, and impulsivity represent significant predictors and have effects on suicidal risk. Regarding our first hypothesis, the analyses suggest that people with greater depressive symptoms, feelings of hopelessness, and impulsivity are at greater risk of attempts and, thus, suicidal risk. These findings are consistent with the results of other studies in which lack of premeditation or difficulties in planning before making decisions, depressive symptoms, and hopelessness have been found to be direct predictors of suicide attempts in young people (Valderrama & Miranda, 2017; Yen et al., 2009).

However, when analyzing the direct effects of depression, hopelessness, and impulsivity on suicidal attempts and risk, the model did not show optimal goodness-of-fit indicators (Model 1, Table 4). These results may indicate that although the existence of different significant predictors of suicidal behavior is clear as suggested by several meta-analyses (Ati et al., 2021; González Sancho & Picado Cortés, 2020; Hernández-Bello et al., 2020), these results may vary when analyzing the mechanisms by which these factors, along with others related to interpersonal functioning, interact (Joiner et al., 2009; O’Connor & Nock, 2014). Specifically, the perception of an inability adequately take responsibility for others, coupled with cognitions associated with dysfunctional affective bonds with other people or the social environment, plays a significant role (Van Orden et al., 2010).

This finding is consistent with Wang et al. (2015), who demonstrated that the interaction between depressive symptoms and impulsivity can significantly predict suicide risk. Furthermore, they found that impulsivity moderated the relationship between depression and suicide risk. In our study, impulsivity showed the strongest clear relationships with suicidal risk (*r* = 0.47) and depression (*r* = 0.43), indicating that people with more severe depression tend to be more impulsive and have higher suicidal risk. This is consistent with studies that have analyzed risk factors and mediating effects between impulsivity and suicidal risk (Dvorak et al., 2013; Gómez-Tabares et al., 2020).

This mechanism of interaction effects between depressive symptoms, feelings of hopelessness, and impulsivity with respect to suicidal risk and behavior has been addressed in different research (Javdani et al., 2011; Koyama et al., 2020; Liu et al., 2020; Zhang et al., 2022) and indicates that the mediating effect of impulsivity is an important bridge between the relationships of depression and hopelessness. Regarding our second hypothesis, our study confirmed that those participants with higher suicide risk were those in whom depressive symptoms and hopelessness were mediated by impulsivity traits, in other words, impulsivity plays a mediating role between depression and hopelessness, and such a role increases the effect of both predictors on suicide risk.

In addition, it was shown that the proposed model remains invariant when sex differences (male/female) are taken into account, reinforcing the idea that impulsivity is a strong psychological mechanism mediating the interactions between indicators of depression hopelessness, and suicidal behavior in a young population.

Indeed, the results of suicidal ideation have shown significant indirect effects with risk behavior through impulsivity and low desire to live, but not through hopelessness (Smith & Wells, 2023), which leaves an interesting axis of research given that ideation and risk behaviors are associated with each other through impulsivity and desire to live.

Meanwhile, our findings add to the knowledge so far of the mediating effect of impulsivity with respect to depression and hopelessness on suicidal attempts and risk and we can conclude that high levels of trait impulsivity increase the effect of depression and hopelessness on suicidal behavior, which is consistent with empirical evidence from cross-sectional and longitudinal studies where the mediating role of impulsivity on depression and hopelessness has been studied (Arango-Tobón et al., 2021; Wang et al., 2015). On the other hand, we found that impulsivity is also a mediator in the relationships between depression and suicidal attempts, an aspect that is often related to the association of reiterative death ideation and the presence of suicidal attempts (Sohn et al., 2021).

Now, the correlations we found between impulsivity and suicidal intent are low but significant (*r* = 0.2) and may indicate that impulsivity has a direct effect on suicidal intent that consequently generates higher suicide risk in young people. Findings from studies such as that of Zhang et al. (2022) support the conclusion that individuals exhibiting impulsivity traits increase attempts and overall suicidal risk more. Having depressive symptoms and impulsivity traits maximizes the individual or joint effect of depression and hopelessness on suicidal behavior.

Although the data collected allowed us to test the two central hypotheses of our study, it is important to point out some limitations. 1) The cross-sectional design and the unknown predictive validity of the probability of suicide in the sample reduces the explanatory capacity of our model on the mediating role of impulsivity in populations of young university students. Although there are advances in predictive models related to impulsivity, ideation, and suicidal risk (Beach et al., 2022), new studies that increase the explanatory capacity of statistical models that involve impulsivity as a mediator of suicidal risk are worthwhile. In this regard, it is necessary to consider that the study did not consider specific clinical aspects of physical and mental health as inclusion criteria, which may be expanded for analysis in future studies.

Indeed, as proposed by Joiner (2005), Ma et al. (2016), Baertschi et al. (2017), and Chu et al. (2017), it is also necessary to examine other interpersonal variables to determine whether they moderate or mediate the relationship with suicide risk (Chu et al., 2016; O’Connor & Portzky, 2018). While the study's results show promise in explaining how impulsivity mediates the effect of depression and hopelessness on suicide risk, further exploration of the relationship with interpersonal variables in future studies may enhance the predictive scope for suicidal behavior (Joiner et al., 2009; Puzia et al., 2014).

It is therefore suggested that these findings should be interpreted carefully and that more longitudinal studies are needed to provide predictive evidence of suicide and of the mediating or moderating role of impulsivity. 2) The use of self-report measures to establish depressive symptoms, hopelessness, and impulsivity may represent a bias in the information analyzed since self-reports are based on self-awareness and recall of behavioral patterns related to suicidal attempts and risk. 3) The sample is composed of young university students, and it is not clear that our results can be generalized to other populations, so it is suggested that this study could be replicated in other clinical or non-clinical samples. It would be desirable for future studies to analyze not only trait impulsivity but also impulsivity as a state, in order to analyze the effect that impulsivity as a psychological or neuropsychological characteristic may have on depression and hopelessness. Finally, it is suggested, as proposed by Anvar et al. (2022), to analyze impulsivity in relation to the severity of emotional fluctuation, uncontrollability, dynamic course, and affective and cognitive precursors of suicidal ideation and suicide attempts.

## Supplementary Materials

The Supplementary Materials contain the following items (for access, see Gómez-Tabares et al., 2025S):

- Supplementary Table 1: Indicators of mental health events (suicide risk and attempt, depression, hopelessness, and impulsivity)
- Supplementary Table 2: Sex differences in suicide risk and attempt, depression, hopelessness, and impulsivity

Gómez-TabaresA. S.
Arango-TobónO. E.
NúñezC.
Zapata LesmesG. A.
 (2025S). Supplementary materials to "The effect of depression and hopelessness on suicidal risk in young people: The mediating role of impulsivity"
[Supplementary tables]. PsychOpen. 10.23668/psycharchives.16212
PMC1215222440510540

## Data Availability

Data supporting the conclusions of this study are available upon reasonable request to the corresponding author. Data are not publicly available due to ethical restrictions on informed consent.

## Reporting Guidelines

The JARS-Quant guidelines for non-experimental quantitative research were followed in this study.

## Preregistration

This study is not pre-registered. The study was registered at the Universidad Católica Luis Amigó, Medellín, Colombia [05020299123].
